# Physical fitness cognition, assessment, and promotion: A cross-sectional study in Taiwan

**DOI:** 10.1371/journal.pone.0240137

**Published:** 2020-10-06

**Authors:** Te-Wei Ho, Hsing-Hua Tsai, Jui-Fen Lai, Sue-Min Chu, Wan-Chung Liao, Han-Mo Chiu

**Affiliations:** 1 Department of Surgery, College of Medicine, National Taiwan University, Taipei, Taiwan; 2 Health Management Center, National Taiwan University Hospital, Taipei, Taiwan; 3 Department of Internal Medicine, College of Medicine, National Taiwan University, Taipei, Taiwan; University of Zurich, SWITZERLAND

## Abstract

**Introduction:**

Many health organizations have promoted the importance of the health-related benefits of physical fitness and physical activity. Studies have evaluated effective public health practice aiming to understand the cognition of physical activity among youths and adolescents. However, studies investigating the level of cognition and knowledge of physical fitness among Asian adults are lacking.

**Purpose:**

This study aimed to investigate the self-awareness level of physical fitness and exercise prescription and the demand for physical fitness assessment among Taiwanese adults.

**Methods:**

In January–July 2019, a cross-sectional anonymous survey was conducted using Research Electronic Data Capture to gather data on demographic data, cognition investigation of physical fitness and exercise prescription, cognitive test of physical fitness and exercise prescription, and demand for physical fitness assessment.

**Results:**

The questionnaire was answered by 200 respondents. The rating for cognition investigation of physical fitness was 2.63–3.13 (unclear to mostly clear) and for exercise prescription was 2.05–2.76 (unclear) (rated on a 5-point Likert scale). Results show that lack of awareness was highest for health-related physical fitness, exercise prescription, and exercise progress planning. 98% of subjects did not know the latest recommended guidelines for physical activity, despite most agreeing that physical fitness and exercise are good for health. Most subjects (72%) indicated a willingness to accept self-pay service for physical fitness assessments.

**Conclusions:**

This is the first study to report on the demand for cognition, assessment, and promotion of physical fitness among Taiwanese adults. The study shows that the subjects widely lack knowledge in the cognition of physical fitness and exercise prescription. Furthermore, a self-pay service for the physical fitness assessment and individualized exercise prescription were acceptable to most subjects, especially those undergoing regular health examinations. The findings are encouraging and will aid support for health organizations and professionals in the development and management of promotion strategies on health-related physical fitness in preventive medicine and health promotion.

## Introduction

Physical fitness, a state of good health and strength, which is a comprehensive ability, is a multifactorial concept covering five components: muscular strength, muscular endurance, flexibility, body composition, and especially cardiorespiratory fitness [1, 2], all of which are related to the body’s ability to adapt to life and the environment [3, 4]. In general, individuals with better physical fitness have better energy and adaptability to carry out daily tasks or work, especially physical activity or exercise [5]. Moreover, a number of evidence-based studies have consistently indicated that moderate physical and cardiorespiratory fitness could create a protective effect for some cancers [6] and reduce the risks of cardiovascular diseases and metabolic syndrome [7, 8], especially risks of all-cause and cardiovascular mortality [9–15]. Health organizations such as the American College of Sports Medicine (ACSM), in collaboration with the Centers for Disease Control and Prevention (CDC), started to propose a guideline of physical activity in 1995 to increase public awareness of the importance of the health-related benefits of physical activity [16]. The latest guidelines state that to achieve health benefits in adults aged >18 years, one must engage in at least 150 minutes of moderate-intensity exercise or 75 minutes of vigorous exercise per week, or an equivalent combination of moderate and vigorous exercise [16, 17]. To encourage students to adopt lifelong exercise habits, in 1999, the Ministry of Education in Taiwan initiated a project entitled “Physical Fitness 333 Plan,” where students (aged 6–15 years) were encouraged to exercise for 30 minutes at least three times per week, with heart rate during exercise reaching at least 130 bpm or higher [18]. This protocol, however, is proposed mainly for students and does not take age, health profile, and other issues in the general population into account, making it unsuitable for elderly people or patients with diseases. Most people in Taiwan still consider it as the latest exercise guideline for the general population around the world, despite that the main purpose of the 333 Plan is to advocate establishing regular exercise habits; also, people usually do not know the difference between moderate-intensity and vigorous exercise and the intensity of different forms of physical activity. Furthermore, many reports have indicated that to achieve high compliance and achievement of goals, plans for fitness-related activities must be individualized and personalized according to the individual’s specified purpose and his/her unique needs and interests [19–23]. In the past decades, the effects of physical fitness on quality of life, health, and life expectancy have been proven by number of studies and reports. To the best of our knowledge, cognition of physical fitness and physical activity recommendations are pivotal prerequisites for actual change in behavior and action [24, 25]. Significant associations between cognition of physical activity recommendations and activity behavior have been well documented [26–28], and educational exposure to physical activity recommendations had a significantly positive effect on the improvement of activity behavior [29]. Another study also reported that increasing cognition of physical activity recommendations may be an effective promotional strategy to develop and augment intentions to engage in physical activity [30]. A great deal of previous researches has been directed at understanding the cognition of physical activity among youths and adolescents [29, 31–33]. However, there is a lack of studies investigating the level of cognition and knowledge of physical fitness in Asian adults. To address these issues, this study aimed to investigate the cognition of physical fitness and exercise prescription and the demand for physical fitness assessment among adults in Taiwan.

## Materials and methods

### Participants and data collection

In this cross-sectional study, an anonymous questionnaire was used for the survey, which was conducted from January 2019 to July 2019 at the Health Management Center of the National Taiwan University Hospital (NTUH) and public websites. We put up flyers with an introduction and a website link for a questionnaire investigation on these public study fields. No inclusion and exclusion criteria were defined for the subject recruitment. When participants learned about the recruitment, they joined voluntarily and were assured confidentiality and that they were free to withdraw from the survey at any time. A double-checked button was used to signify that the participant was done answering the questionnaire. No compensation was provided to the participants for their time. Of 268 responses during the study period, 68 questionnaires with missing data were excluded from the study. A total of 200 individuals voluntarily answered the anonymous questionnaire, along with complete records, which were included in this study for further data analysis. The institutional review board of the NTUH approved the study protocol (201908044RINC).

### Survey development and procedures

Research Electronic Data Capture (REDCap), a web-based platform hosted at the NTUH, was used to develop, collect, and manage the anonymous questionnaire. REDCap, which is a compatibility, scalability, and security data collection tool, along with built-in analysis tools was developed at Vanderbilt University in 2004 for the purpose of clinical research [34]. REDCap was designed to meet Health Insurance Portability and Accountability Act compliance standards. REDCap is widely used in the academic research community: currently, it has 3,719 partner institutions in 131 countries, supports more than 735,000 projects with 1 million end-users, and has been cited in 7,136 journal articles [35].

The custom-designed REDCap online survey can be accessed and filled out by participants using a smartphone, tablet, or computer with internet connection. The Hypertext Transfer Protocol Secure (HTTPS) protocol for secure internet communication transmits the feedbacks automatically into the hospital database. NTUH maintains the research database, and to access the management tool, researchers must enter their username and password. The hospital portal system, along with one‐time password from email or Google Authenticator Application, validates the login session. User behavior and manipulation on the platform are tracked as log messages into database. The data format of REDCap fulfills the Clinical Data Interchange Standards Consortium standard. The researchers could use automatic export procedures for seamless data downloads including raw data and variable schema to common data types such as CSV, Excel, SPSS, SAS, R, Stata, and XML.

### Questionnaire design

A research team that specialized in exercise physiology, medicine, and health management developed the questionnaire material in a roundtable conference for fulfilling the need of investigation (the study questionnaire is presented in S1 File). An introduction paragraph in the questionnaire stated the purpose of the study, that is, to investigate the cognition and demand of the people for physical fitness. To address the purpose of this study, survey questions broadly fell into the following categories: basic demographic data (four items), cognition investigation of physical fitness (nine items), cognition investigation of exercise prescription (seven items), cognitive test of physical fitness and exercise prescription (nine items), and demand for physical fitness assessment (six items). Content validity of the study questionnaire has been verified, with a mean Cronbach’s *α* of 0.912 (minimum = 0.905; maximum = 0.922) for cognition investigation of physical fitness and 0.920 (minimum = 0.911; maximum = 0.926) for cognition investigation of the exercise prescription, both of which indicate excellent internal consistency. All sensitive information, such as name, identification number, telephone number, email, and Internet Protocol address (IP address) of the electrical devices were not collected in this study. A 5-point Likert scale (1 = very unclear, 2 = unclear, 3 = mostly clear, 4 = clear, and 5 = very clear) was used to rate the cognition items. Higher scores indicate positive cognition. The true-or-false tests were presented as positive and negative statements. Finally, the questionnaire was divided into five pages on the REDCap online survey, with each page having an intuitive interface for the validation of data entry without any missing item. The study team conducted complete checks for content typo and interactive buttons before starting the survey. Although this was an anonymous questionnaire, the participants still had to click a button to accept and continue with the survey at the beginning of the questionnaire.

### Outcome and demographic variables

We calculated the average score of the cognition items based on the 5-point Likert scale and the accuracy of true-or-false tests as study outcomes, both of which represent the levels of cognition of physical fitness and exercise prescription. We assessed the following demographic data: gender, age (20−29, 30−39, 40−49, 50−59, 60−69, and ≥70 years), undergoing regular health examination and frequency (once every year, once every 2 years, once every 3 years, and casual). In addition, the following data were gathered to determine the demand for physical fitness assessment: factors that affected the choose of a physical fitness test service, willingness to accept self-funded physical fitness tests and the acceptable price, having undergone a physical fitness test in the past 5 years, and having received guidance related to exercise prescriptions in the past. Furthermore, we evaluated the distribution of the demand for physical fitness assessment stratified by subjects with and without regular health examinations.

### Data analysis

To calculate numbers and percentages for all question items among respondents, descriptive statistics were initially performed in REDCap. Figures were generated using Microsoft Office Excel 2019. The results of cognition items are presented as means with 95% confidence intervals (CIs). Further statistical analysis was conducted using SPSS software (Version 23; IBM Corp., Armonk, NY). Chi-square test or Fisher exact test was used for comparisons of willingness between men and women among different age groups, as appropriate. Statistical significance was set at *P* < 0.05. Reliability analysis was evaluated by Cronbach’s alpha for the content validity of questionnaire.

## Results

We collected data from a total of 200 respondents (114 men and 86 women) for further analysis. Age distributions, in order, were as follows: 40–49 years (n = 84, 42%), 50–59 years (n = 45, 22.5%), and 30–39 years (n = 42, 21%). Most subjects were educated at the university (n = 104, 52%) and postgraduate or above (n = 88, 44%) level. Seventy-nine percent of the subjects receive regular health examinations and usually undergo examination once a year (*n* = 64, 40.5%) (distribution of regular health examination stratified by gender is presented in S2 File). Table 1 summarizes the subject characteristics.

**Table 1:** Characteristics of subjects (N = 200).

Characteristic	Number	(%)
Gender		
Male	114	(57.0%)
Female	86	(43.0%)
Age		
20 ~ 29	13	(6.5%)
30 ~ 39	42	(21.0%)
40 ~ 49	84	(42.0%)
50 ~ 59	45	(22.5%)
60 ~ 69	12	(6.0%)
≥ 70	4	(2.0%)
Education		
Senior high school	8	(4.0%)
University	104	(52.0%)
Postgraduate or above	88	(44.0%)
Regular health examination		
Yes	158	(79.0%)
Once every year	64	(40.5%)
Once every two years	61	(38.6%)
Once every three years	15	(9.5%)
Casual	18	(11.4%)
No	42	(21.0%)

### Cognition investigation of physical fitness and exercise prescription

**Fig 1:**
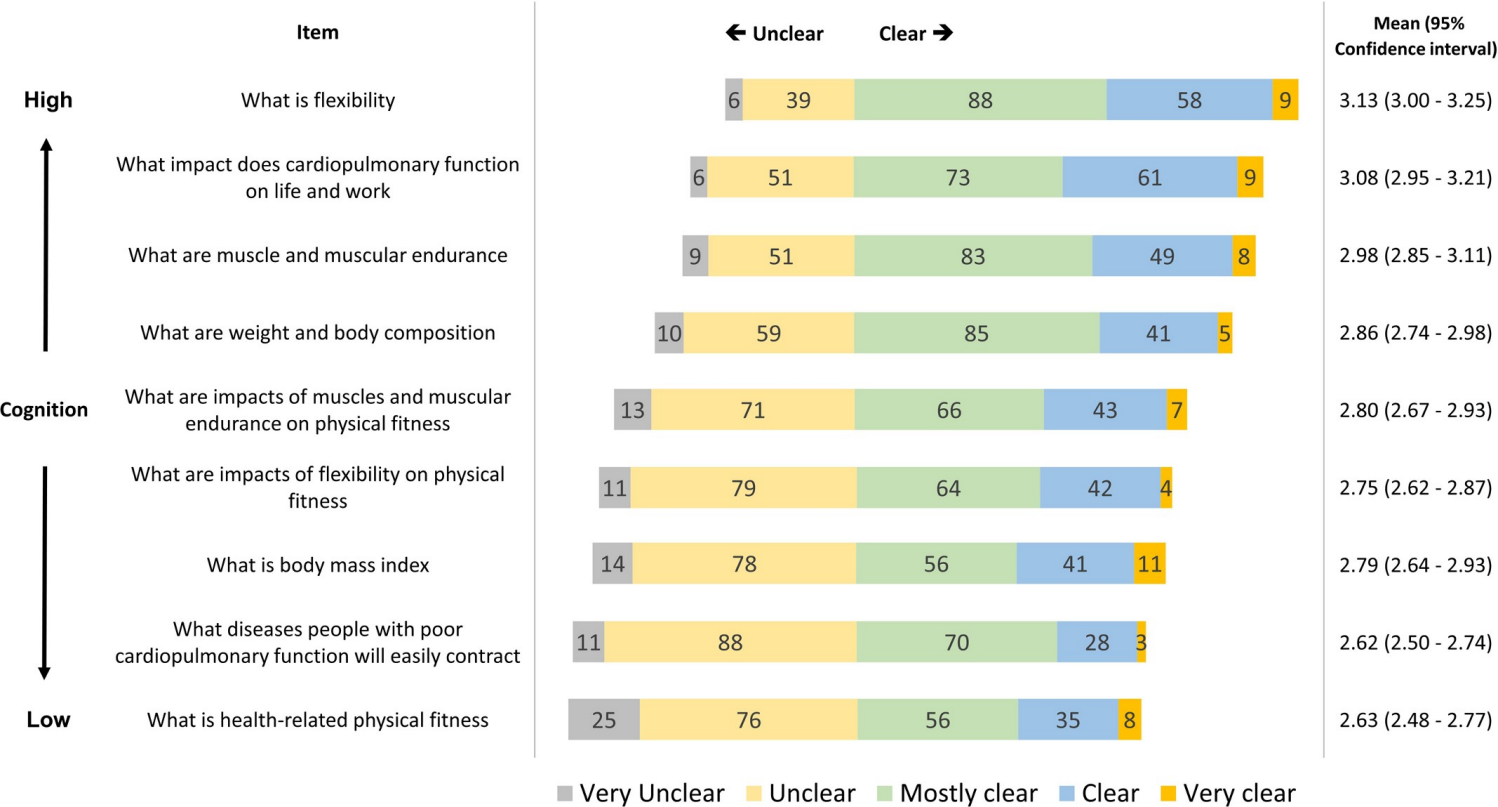
Results of the investigation on cognition of physical fitness, along with rank ordering, according to distribution of responses as “very unclear” and “unclear”.

With regard to cognition of physical fitness, subjects’ replies using the 5-point Likert scale ranged from 2.63 to 3.13, indicating that the general cognition of physical fitness is “unclear” to “mostly clear.” Higher scores were found for cognition for flexibility (5-point Likert mean, 3.13; 95% CI, 3.00–3.25), impact of cardiopulmonary function on life and work (5-point Likert mean, 3.08; 95% CI, 2.95–3.21), and muscle and muscular endurance (5-point Likert mean, 2.98; 95% CI, 2.85–3.11). Physical fitness items that had the highest response rates of “very unclear” and “unclear” are health-related physical fitness (*n* = 101, 50.5%), what diseases people with poor cardiopulmonary functions will easily contract (*n* = 99, 49.5%), and body mass index (*n* = 92, 46%). Results of the investigation of cognition of physical fitness in this study are presented in Fig 1, along with rank ordering according to the distribution of responses of “very unclear” and “unclear.”

**Fig 2:**
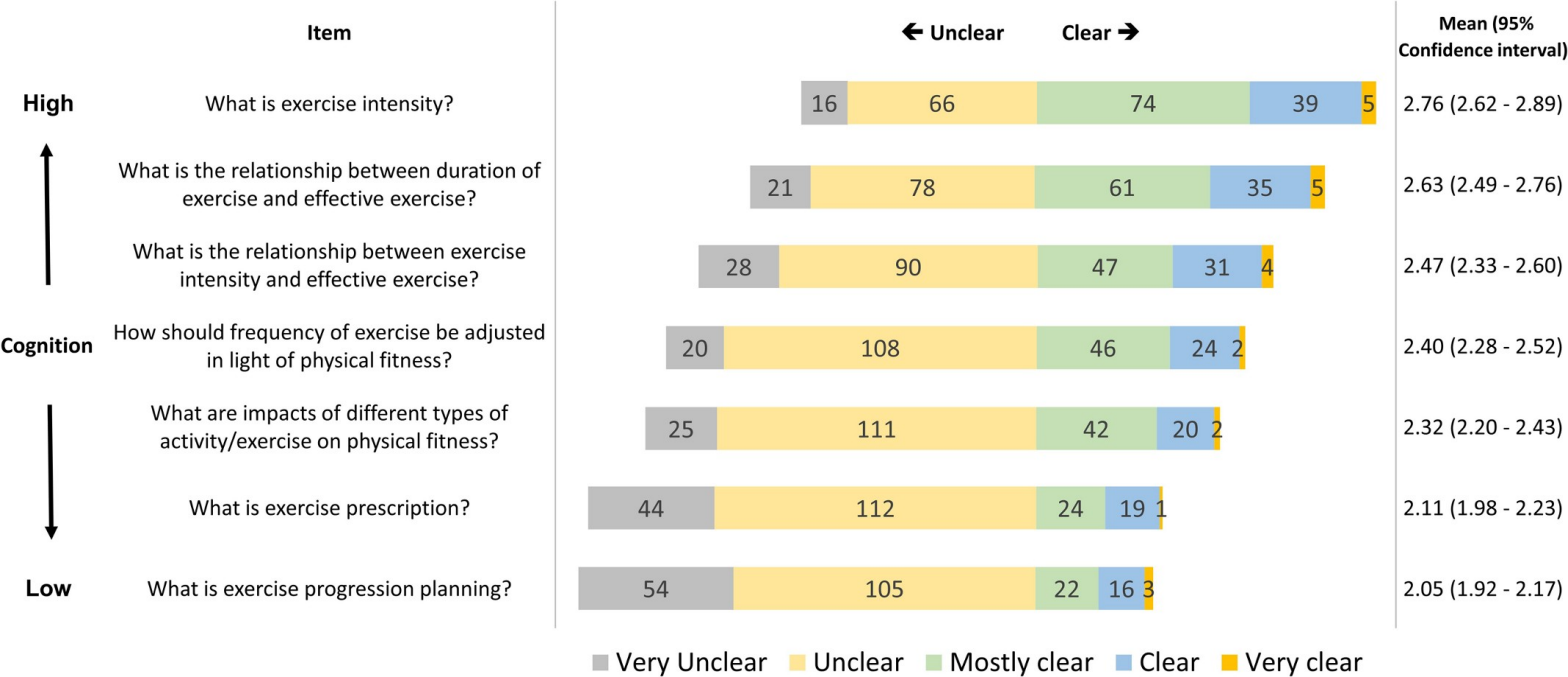
Results of the investigation on cognition of exercise prescription, along with rank ordering, according to distribution of responses as “very unclear” and “unclear”.

For cognition of exercise prescription, the results show that participant responses on the 5-point Likert ranged from 2.05 to 2.76, indicating that the cognition of exercise prescription among subjects is, in general, “unclear.” Results show that cognition for exercise intensity (5-point Likert mean, 2.76; 95% CI, 2.62–2.89), the relationship between duration of exercise and effective exercise (5-point Likert mean, 2.63; 95% CI, 2.49–2.76), and the relationship between exercise intensity and effective exercise (5-point Likert mean, 2.47; 95% CI, 2.33–2.60) had higher ratings. The items with the highest rates of “very unclear” and “unclear” ratings are exercise progression planning (*n* = 159, 79.5%), exercise prescription (*n* = 156, 78%), and impacts of different types of activity/exercise on physical fitness (*n* = 136, 68%). Results of the investigation on cognition of exercise prescription, along with rank ordering according to the distribution of “very unclear” and “unclear” responses, are presented in Fig 2.

### Cognitive test of physical fitness and exercise prescription

**Fig 3:**
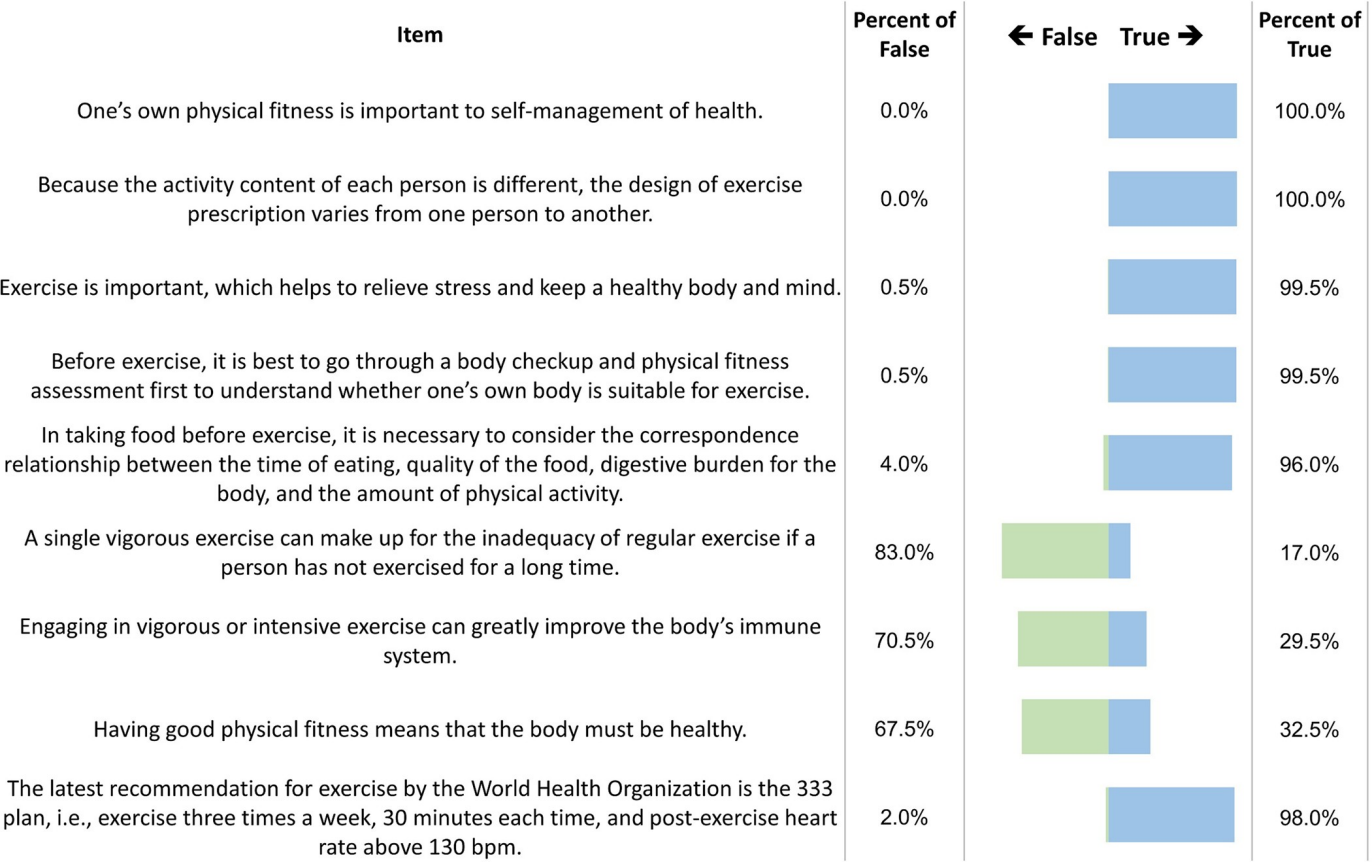
Results of cognitive test of physical fitness, along with rank ordering, according to accuracy.

With regard to cognitive test of physical fitness, results indicate that all subjects know (*a*) that understanding one’s own physical fitness is important to self-management of health and (*b*) that, because the activity content of each person is different, the design of exercise prescription varies from one person to another. However, results show that subjects lack knowledge in the following areas: (1) a single vigorous exercise can make up for the inadequacy of regular exercise if a person has not exercised for a long time (accuracy rate, 83%); (2) engaging in vigorous or intensive exercise can greatly improve the body’s immune system (accuracy rate, 70.5%); and (3) having good physical fitness means that the body must be healthy (accuracy rate, 67.5%). In addition, although the 333 Plan had already been considered out-of-date, up to 98% of respondents consider it as the latest exercise recommendations. Fig 3 shows the results of cognitive test for physical fitness along with rank ordering according to accuracy.

### Demand for physical fitness assessment

**Fig 4:**
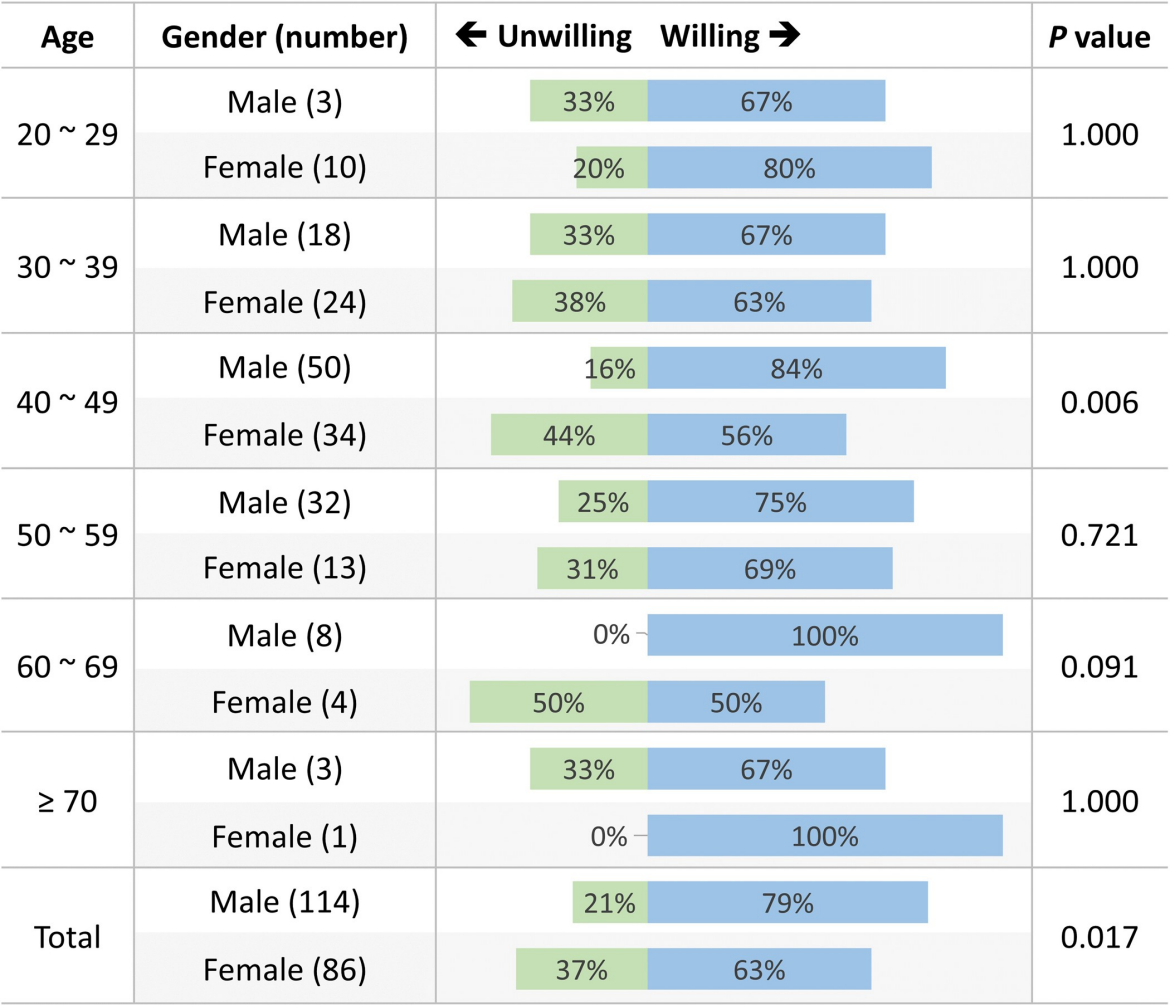
Distribution of willingness to accept a self-pay service for physical fitness assessment, according to sex and different age groups.

**Fig 5:**
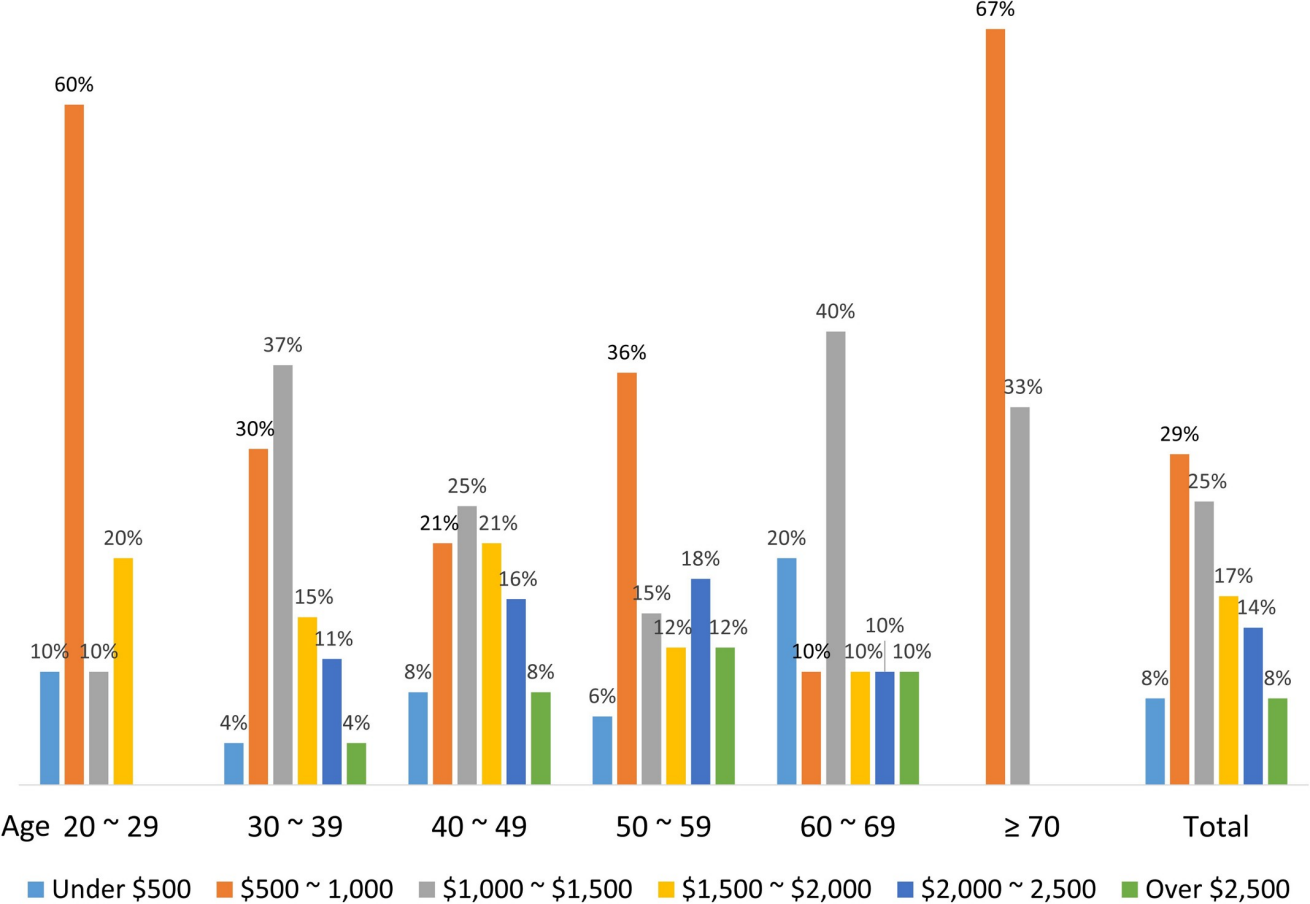
Acceptable price ranges for self-pay service of physical fitness, according to different age groups (in New Taiwan Dollar [NTD], 1 US$ = 30 NTD).

Overall, 144 (72%) subjects are willing to accept a self-pay service for physical fitness assessments, with more men (79%) than women (63%) agreeing to this (*P* = 0.017). In the comparison of sexes in each age category, significant differences were found only in subjects between 40 and 49 years (*P* = 0.006; men, 84%; female, 56%). The distribution of willingness to accept a self-pay service for physical fitness assessment by sex, according to different age groups, is displayed in Fig 4. With regard to the acceptable price for a single physical fitness assessment service, the price ranges acceptable according to participant responses were as follows (in order, in NT $): $500–$1,000, *n* = 42 (29%); $1,001–$1,500, *n* = 36 (25%); $1,501–$2,000, *n* = 24 (17%); $2,001–$2,500, *n* = 20 (14%); under $500, *n* = 11 (8%); and greater than $2,500, *n* = 11 (8%). Fig 5 illustrates the distribution of expected price for self-pay service of physical fitness according to age groups.

With regard as to why participants were willing to accept a self-pay service, the main reasons were the provision of (1) individualized exercise prescription according to physical fitness examination results (*n* = 160, 80%), (2) individualized report according to the physical fitness examination results (*n* = 151, 75.5%), and (3) reasonable and achievable health-related physical fitness goals (*n* = 144, 72%). In contrast, the underlying reasons for not choosing a self-pay service for physical fitness assessment are the following: (1) does not want to pay for physical fitness tests (*n* = 27, 13.5%), (2) no time (*n* = 14, 7%), (3) feeling that one is healthy (*n* = 13, 6.5%), and (4) does not know any place that provides physical fitness examinations (*n* = 12, 6%).

## Discussion

This article presents the first findings regarding the cognition of physical fitness and exercise prescription and the demand of physical fitness assessment among adults in Taiwan. The present experimental results show ranges of 2.63–3.13 for cognition investigation of physical fitness and 2.05–2.76 for exercise prescription, rated on a 5-point Likert scale, indicating that the subjects’ cognition level of physical fitness is between “unclear” to “mostly clear” and exercise prescription is “unclear.” In addition, results show that the items health-related physical fitness, exercise prescription, and exercise progress planning were the areas that most participants lack an awareness of, all of which are terms unfamiliar to the public. Although approximately all subjects agreed that physical fitness and exercise are good for the health, up to 98% of subjects do not know the latest recommended guidelines for physical activity, a finding that is in accordance with previous large-scale studies. In a cross-sectional national survey (n = 4,281) conducted in the United States, only 36.1% of US adults had ever received messages regarding government physical activity guidelines from any type of media, of which only 1% were aware that for substantial health benefits, the recommended volume of exercise was at least 150 minutes of moderate-intensity exercise per week [36]. A cross-sectional study in the United Kingdom reported that in 2013, 18% of 1,797 adults accurately recalled the physical activity guidelines, compared with 11% of 2,860 adults in 2007 [37]. Similarly, another national survey conducted on 10,992 adults between December 2013 and September 2014 in the United Kingdom showed that only 15% of adults accurately reported the recommendation guideline on physical activity [38]. Despite many organizations such as the ACSM, CDC, American Heart Association (AHA), and World Health Organization (WHO) focus on global health and widely promote the benefits and guidelines of physical fitness, keeping the right cognition in mind is still difficult for most people, especially putting it into action. In addition, several studies have reported that the multitude of physical activity recommendations released over the years may have caused confusion among people [39–41]. This once again highlights the importance of promoting the right knowledge.

In Taiwan, life expectancy for the general population is 80.4 years, with rates of 77.3 and 83.7 years among men and women, respectively [42]. With regard to the proportionate increase in the elderly population each year, the main purpose of health organizations in Taiwan is to advocate “Healthy Life Expectancy,” which promotes initiatives that focus on healthy lifestyle, including no tobacco, healthy diet, stress relief, regular exercise, and physical fitness. The first step for medical and health care institutions with regard to primary prevention is to make individuals aware and knowledgeable of physical fitness, which is necessary for making health-related decisions [43]. In the past, assessment and counseling for physical activity were generally not a focus of most health professionals’ training programs [44, 45]. A scientific statement from AHA described lifestyle counseling during medical school training and indicated a low percentage of primary care providers who discuss lifestyle and physical activity issues with patients during clinic visits [46]. A recent study showed that most physicians provided counseling and prescription for exercise in only less than 10% of appointments [47]. In a cohort study for patients with obesity, primary care providers provided counseling on exercise and physical activity in only 20% of office visits [48]. However, most of subhealth people and patients, especially individuals who have an inactive lifestyle or who are in a particularly urgent need of moving more, need recommendations or interventions for activity and exercise. All medical practitioners, including physicians, nurses, physical and occupational therapists, exercise physiologists, dieticians, and any healthy lifestyle providers, should routinely integrate the concept of physical fitness and exercise into their practice. Despite the possibility that assessment and intervention will vary greatly across them, attempts to ask several simple health-related fitness questions in counseling should at least be made; for example, if the patient reveals characteristics of inactive activity or a completely sedentary lifestyle, the importance of physical activity should be emphasized. In addition, medical professionals should appropriately refer patients to other members of the multidisciplinary team for further health-related fitness assistance. Previous studies also mentioned that a more attractive approach to improve healthy lifestyle behaviors is health care practice across multidisciplinary professionals [49, 50].

For public health organizations such as government agencies and nongovernmental organizations, the main purpose of health dissemination is carried out through multiple channels to raise general public awareness on the importance of the health-related benefits of physical fitness, especially in physical activity and moderate-intensity exercise. In addition, physical inactivity and sedentary lifestyle are global adverse health effects, which have been identified to contribute to the burden of many chronic diseases and premature mortality [51–53]. The concept of “exercise is medicine” or “movement is medicine” (i.e., taking more daily steps as possible, sitting as little as possible, and having regular exercise habit) should be more widely advocated in developing individual healthful living behavior. Both physical educators and health promoters should assume leadership to be a model for the public and apply effective public health campaign and behavioral strategies, such as social support and enrichment sport activities, to foster recommendation adherence [30, 54]. It has also been reported in recent studies that attractive environments could influence physical activity behavior and the health profile of community residents [55–58].

In this study, a self-pay service for physical fitness assessments was acceptable to most subjects (72%). The most acceptable price for a self-pay service for physical fitness assessments was approximately $500−$1,500 New Taiwan Dollars (NTDs) (30 NTDs = 1 US dollar). In Taiwan, the prices of visiting a clinic, undergoing a labor health examination, and undergoing a health examination package generally are approximately $150−$350, $1,000−$1,500, and $20,000−$150,000, respectively. Providing this augmentation service in health examination packages would require the setting of an affordable price. Moreover, compared with subjects who did not go for regular physical examinations, individuals who undergo regular health examinations were more accepting of a self-pay service for physical fitness assessments (57% vs. 76%; *P* = 0.02; S3 File). More importantly, 92.4% of subjects who go for regular health examinations did not receive any physical fitness evaluation in the past 5 years (S4 File). This indicates that the identification of any possible signs or symptoms of a medical condition is the main focus of current regular health examinations, and the concept of health-related physical fitness is still not integrated into preventive healthcare services. The preliminary results of this study indicate that people are eager to receive and know their health-related physical fitness profile and to obtain further personalized suggestions and exercise prescriptions. Generally, medical practitioners are often swamped with clinical responsibilities that they do not have enough time to conduct a routine assessment, discussion, and plan of physical fitness for patients. Fortunately, with information and communication technologies progressing and developing, facilities with internet of things technology such as wearable devices and smartphones have carried out appropriate approaches, along with objective quantitative data, for measurement and management in physical fitness and physical activity [59–62].

This cross-sectional study had some limitations that should be mentioned. First, because of the limited number of participants, the present findings are limited to represent the ideas of the general population in Taiwan, which may affect the inference to various Asian peoples of different nationalities or ethnicities. Second, most of the responses presented were that of having regular health check-ups and theoretically more concerned about their own health, which may overestimate the demand of physical fitness assessment for adults in Taiwan. Similarly, recruitment at a health care facility and on websites may have increased the possibility of bias in this cross-sectional study. People who were concerned about their health may voluntarily prefer to answer the study questionnaire, which may also result in an over-estimated investigation. In contrast, the level of cognition of physical fitness for adults may be overestimated due the abovementioned issue. Third, some potential confounding factors, such as social-economic level, residential area, marriage status, and health-related status, were not collected in this survey owing to the anonymous nature of the questionnaire. To achieve a discussion from multiple perspectives and perspectives based on further results, future studies should consider designs that include a more personal profile.

Our next goal is to focus on advocating, through multidisciplinary professionals, the importance of self-awareness of health-related fitness to the general public and determine whether the assessment and intervention of physical fitness could actually affect people’s behavior and health condition.

## Conclusion

Our study is the first to report on the urgent demand for cognition, assessment, and promotion of physical fitness among adults in Taiwan. This cross-sectional investigation showed that many subjects lacked cognition of physical fitness and exercise prescription. A self-pay service for the physical fitness assessment and individualized exercise prescription were acceptable to most subjects, especially to individuals who were undergoing regular health examinations. The findings in this study may have pivotal implications for the lack of promotion and implementation of health-related physical fitness plans. Health organizations and professionals can use the insights in this study to aid in the development of strategies, promotion of correct augmented knowledge, and conduct of effective practical guidelines in the fields of preventive medicine and health promotion.

## Supporting information

S1 File Study questionnaire. (DOCX)

S2 File Distribution of regular health examinations stratified by gender. (DOCX)

S3 File Distribution of accepting self-pay service for physical fitness assessments stratified by those who had/did not undergo regular health examinations. (DOCX)

S4 File Distribution of receiving physical fitness evaluation in the past 5 years stratified by those who had/did not undergo regular health examinations. (DOCX)

S1 Checklist PLOS ONE clinical studies checklist. (DOCX)

## Abbreviations

ACSM: American College of Sports Medicine
CDC: Centers for Disease Control and Prevention
NTUH: National Taiwan University Hospital
REDCap: Research Electronic Data Capture
HTTPS: Hypertext Transfer Protocol Secure
IP address: Internet Protocol address
Cis: confidence intervals
AHA: American Heart Association
WHO: World Health Organization
NHI: National Health Insurance
