# Supplementary material for: Physical fitness cognition, assessment, and promotion: A cross-sectional study in Taiwan
Source: PLoS One. 2020 Oct 6;15(10):e0240137. doi: 10.1371/journal.pone.0240137 (PMC7537908; doi:10.1371/journal.pone.0240137)
Supplement: S1 File — (DOCX) [file pone.0240137.s001.docx]

**Supplement 1.** Study questionnaire

**Statement of Questionnaire Development**

**This questionnaire was developed in a roundtable conference for fulfilling the need of investigation by a research team that specialized in exercise physiology, medicine, and health management. Listed following are the main developers and their brief introduction. They have approved the questionnaire into present study and agree with submission.**

1. **Dr. Hsing-Hua Tsai:** **She was graduated and got her PhD degree from the Graduate Institute of Rehabilitation Science at the Chang Gung University in Dec 2016. Her current research interests include exercise medicine, physiology of exercise, and fitness & health.**
2. **Prof. & Dr. Han-Mo Chiu: He is currently the clinical professor of National Taiwan University and chief of Health Management Center of National Taiwan University Hospital. He is also the deputy secretary general of Gastroenterological Society of Taiwan and Councilor member of Digestive Endoscopy Society of Taiwan. His research expertise includes early colon cancer-diagnosis and treatment, colorectal cancer screening, health management, and preventive medicine.**

**I. Basic demographic**

1. Gender:
   - Male □ Female
2. Age:

□ 20 ~ 29 □ 30 ~ 39 □ 40 ~ 49 □ 50 ~ 59

□ 60 ~ 69 □ ≥ 70

1. Education level:

□ Illiterate □ Elementary school □ Junior high school

□ Senior high school □ University □ Postgraduate or above

□ Others (please specify): ___________________

1. Do you have regular health examination?

□ Yes, frequency:

□ Once every year □ Once every two years □ Once every three years

□ Casual

□ No

**II. Cognition investigation of physical fitness**

| Item | Very Unclear | Unclear | Mostly clear | Clear | Very clear |
| --- | --- | --- | --- | --- | --- |
| What is health-related physical fitness? |  |  |  |  |  |
| What impact does cardiopulmonary function on life and work? |  |  |  |  |  |
| What diseases people with poor cardiopulmonary function will easily contract? |  |  |  |  |  |
| What are muscle and muscular endurance? |  |  |  |  |  |
| What are impacts of muscles and muscular endurance on physical fitness? |  |  |  |  |  |
| What is flexibility? |  |  |  |  |  |
| What are impacts of flexibility on physical fitness? |  |  |  |  |  |
| What are weight and body composition? |  |  |  |  |  |
| What is body mass index? |  |  |  |  |  |

**III. Cognition investigation of exercise prescription**

| Item | Very Unclear | Unclear | Mostly clear | Clear | Very clear |
| --- | --- | --- | --- | --- | --- |
| What is exercise prescription? |  |  |  |  |  |
| What is exercise intensity? |  |  |  |  |  |
| What is the relationship between exercise intensity and effective exercise? |  |  |  |  |  |
| What is the relationship between duration of exercise and effective exercise? |  |  |  |  |  |
| How should frequency of exercise be adjusted in light of physical fitness? |  |  |  |  |  |
| What are impacts of different types of activity/exercise on physical fitness? |  |  |  |  |  |
| What is exercise progression planning? |  |  |  |  |  |

**IV. Cognitive test of physical fitness and exercise prescription**

| Question | TRUE | FALSE |
| --- | --- | --- |
| The latest recommendation for exercise by the World Health Organization adopts the 333 plan, i.e., exercise three times a week, 30 minutes each time, and post-exercise heart rate above 130 bpm. |  |  |
| Exercise is important, which helps to relieve stress and keep a healthy body and mind. |  |  |
| Having good physical fitness means that the body must be healthy. |  |  |
| Understanding one’s own physical fitness is important to self-management of health. |  |  |
| Before exercise, it is best to go through a body checkup and physical fitness assessment first to understand whether one’s own body is suitable for exercise. |  |  |
| Designs of exercise prescription vary from one person to another, as the activity content of each person is different. |  |  |
| If a person has not exercised for a long time, a single vigorous exercise can make up for the inadequacy of regular exercise. |  |  |
| Engaging in vigorous or intensive exercise can greatly improve the body’s immune system. |  |  |
| In taking food before exercise, it is necessary to consider the correspondence relationship between the time of eating, quality of the food, digestive burden for the body, and the amount of physical activity. |  |  |

**V. Demand for physical fitness assessment**

1. Which factor(s) will affect your willingness to choose a physical fitness test service? (Can choose more than one):

□ New and rigorous evaluation methods and tools

□ Individualized report according to the physical fitness examination results

□ Individualized comparison of historical data

□ Comparison between individual measurement data and data of the public

□ Individualized exercise prescription according to physical fitness examination results

□ Provide reasonable and achievable health-related physical fitness goals

□ Integrate clinical data to assess the conditions of health progression

□ Prices of service

□ All of the above

1. Are you willing to accept self-funded physical fitness tests?

□ Yes. Which do you regarded as the acceptable price for a single physical fitness test and assessment service?

□Under $500 □$500-$1,000 □$1,000-$1,500 □$1,500-$2,000

□$2,000-2,500 □Over $2,500

□ No

1. The main reasons for not willing to choose a physical fitness test service.
   - Feeling that one is healthy.
   - Feeling that physical fitness tests are not of much help to health.
   - Does not want to pay for physical fitness tests.
   - Have done a physical fitness test in the past.
   - Does not know any place that provides physical fitness examinations.
   - No time.
2. In the past five years, have you had a complete, professional physical fitness test?
   - Yes □ No
3. Have you received guidance relating to exercise prescriptions in the past?
   - Yes □ No
4. Do you have any recommendations regarding physical fitness assessments and exercise prescriptions? (Optional) ______________________________
