# Supplementary material for: Physical fitness cognition, assessment, and promotion: A cross-sectional study in Taiwan
Source: PLoS One. 2020 Oct 6;15(10):e0240137. doi: 10.1371/journal.pone.0240137 (PMC7537908; doi:10.1371/journal.pone.0240137)
Supplement: S2 File — (DOCX) [file pone.0240137.s002.docx]

**Supplement 2.** Distribution of regular health examinations **s**tratified by gender

| Age  Regular health examination | 20 ~ 29 | | 30 ~ 39 | | 40 ~ 49 | | 50 ~ 59 | | 60 ~ 69 | | ≥ 70 | | number |
| --- | --- | --- | --- | --- | --- | --- | --- | --- | --- | --- | --- | --- | --- |
| Yes | 5 | (3.2%) | 28 | (17.7%) | 68 | (43%) | 42 | (26.6%) | 11 | (7%) | 4 | (2.5%) | 158 |
| Once every year | 2 | (3.1%) | 10 | (15.6%) | 25 | (39.1%) | 17 | (26.6%) | 10 | (15.6%) | 0 | (0%) | 64 |
| Once every two years | 2 | (3.3%) | 9 | (14.8%) | 27 | (44.3%) | 19 | (31.1%) | 0 | (0%) | 4 | (6.6%) | 61 |
| Once every three years | 0 | (0%) | 4 | (26.7%) | 6 | (40%) | 4 | (26.7%) | 1 | (6.7%) | 0 | (0%) | 15 |
| Casual | 1 | (5.6%) | 5 | (27.8%) | 10 | (55.6%) | 2 | (11.1%) | 0 | (0%) | 0 | (0%) | 18 |
| No | 8 | (19%) | 14 | (33.3%) | 16 | (38.1%) | 3 | (7.1%) | 1 | (2.4%) | 0 | (0%) | 42 |

Data shows as number (%).
