# Supplementary material for: Physical fitness cognition, assessment, and promotion: A cross-sectional study in Taiwan
Source: PLoS One. 2020 Oct 6;15(10):e0240137. doi: 10.1371/journal.pone.0240137 (PMC7537908; doi:10.1371/journal.pone.0240137)
Supplement: S3 File — (DOCX) [file pone.0240137.s003.docx]

**Supplement 3.** Distribution of accepting self-pay service for physical fitness assessments stratified by those who had/did not undergo regular health examinations

| Regular health examination | Accepting of a self-pay service for physical fitness assessments | | | | *P* value |
| --- | --- | --- | --- | --- | --- |
|  | Yes (n=158) | | No (n=42) | |  |
| Yes | 120 | (75.9%) | 24 | (57.1%) | 0.020 |
| Once every year | 47 | (39.2%) | - |  | 0.836 |
| Once every two years | 47 | (39.2%) | - |  |  |
| Once every three years | 11 | (9.2%) | - |  |  |
| Casual | 15 | (12.5%) | - |  |  |
| No | 38 | (24.1%) | 18 | (42.9%) |  |

Data shows as number (%).
