# Supplementary material for: Physical fitness cognition, assessment, and promotion: A cross-sectional study in Taiwan
Source: PLoS One. 2020 Oct 6;15(10):e0240137. doi: 10.1371/journal.pone.0240137 (PMC7537908; doi:10.1371/journal.pone.0240137)
Supplement: S4 File — (DOCX) [file pone.0240137.s004.docx]

**Supplement 4.** Distribution of receiving physical fitness evaluation in the past 5 years stratified by those who had/did not undergo regular health examinations

| Regular health examination | Receiving physical fitness evaluation in the past 5 years | | | | *P* value |
| --- | --- | --- | --- | --- | --- |
|  | Yes (n=158) | | No (n=42) | |  |
| Yes | 12 | (7.6%) | 3 | (7.1%) | 1.000 |
| Once every year | 4 | (33.3%) | - |  | 0.396 |
| Once every two years | 7 | (58.3%) | - |  |  |
| Once every three years | 1 | (8.3%) | - |  |  |
| Casual | 0 | (0%) | - |  |  |
| No | 146 | (92.4%) | 39 | (92.9%) |  |

Data shows as number (%).
